# High-Density Porous Graphene Arrays Enable Detection and Analysis of Propagating Cortical Waves and Spirals

**DOI:** 10.1038/s41598-018-35613-y

**Published:** 2018-11-20

**Authors:** Xin Liu, Yichen Lu, Duygu Kuzum

**Affiliations:** Department of Electrical & Computer Engineering, University of California, San Diego, La Jolla, CA 92093 USA

## Abstract

Cortical propagating waves have recently attracted significant attention by the neuroscience community. These travelling waves have been suggested to coordinate different brain areas and play roles in assisting neural plasticity and learning. However, it is extremely challenging to record them with very fine spatial scales over large areas to investigate their effect on neural dynamics or network connectivity changes. In this work, we employ high-density porous graphene microelectrode arrays fabricated using laser pyrolysis on flexible substrates to study the functional network connectivity during cortical propagating waves. The low-impedance porous graphene arrays are used to record cortical potentials during theta oscillations and drug-induced seizures *in vivo*. Spatiotemporal analysis on the neural recordings reveal that theta oscillations and epileptiform activities have distinct characteristics in terms of both synchronization and resulting propagating wave patterns. To investigate the network connectivity during the propagating waves, we perform network analysis. The results show that the propagating waves are consistent with the functional connectivity changes in the neural circuits, suggesting that the underlying network states are reflected by the cortical potential propagation patterns.

## Introduction

Electrophysiological recordings with high spatial resolution are essential for investigating the activity of local neural circuits and cortical potential propagation patterns. Recently, rotating waves and directional potential propagation patterns have been reported in cortical recordings in the human brain during sleep^1–3^ and in epileptic animal models^4,5^. The recorded traveling waves usually consist of either narrow frequency band oscillations, which is the case in human sleep, or wide frequency band energy bursts, which appear in epileptic conditions. On the other hand, in network neuroscience literature, it has also been demonstrated that the functional connectivity of the brain changes during sleep^6–8^ and during seizure development^9,10^. However, it is not clear whether the functional network connectivity changes within the brain regions are reflected or accompanied by the abundant propagating surface potentials. To investigate this, recordings of neural potentials across large areas with high spatial resolution and fidelity are required.

Here, we present a flexible high-density porous graphene microelectrode array specifically designed to provide high spatiotemporal resolution needed for detection of cortical dynamics^11^. Low-impedance porous graphene electrodes record neural activity with high signal-to-noise ratio and enable the detection of cortical patterns with high sensitivity. We perform *in vivo* animal experiments on rodents during which the porous graphene microelectrode array successfully records diverse types of brain activity patterns, including stationary waves, traveling plane waves, and spirals. These different dynamics in surface potentials may imply rich ongoing network activities in the local neuronal circuits. To investigate this, we analyze the recorded cortical potentials (micro-ECoG), explore the spatiotemporal characteristics of different neural activities, and employ a k-means clustering algorithm to identify different propagation patterns during different phases of seizures. Furthermore, we perform network analyses^9^ on the seizures to investigate the relationship between the functional connectivity states of the neural circuits and the propagating cortical waves.

## Results

**Figure 1:**
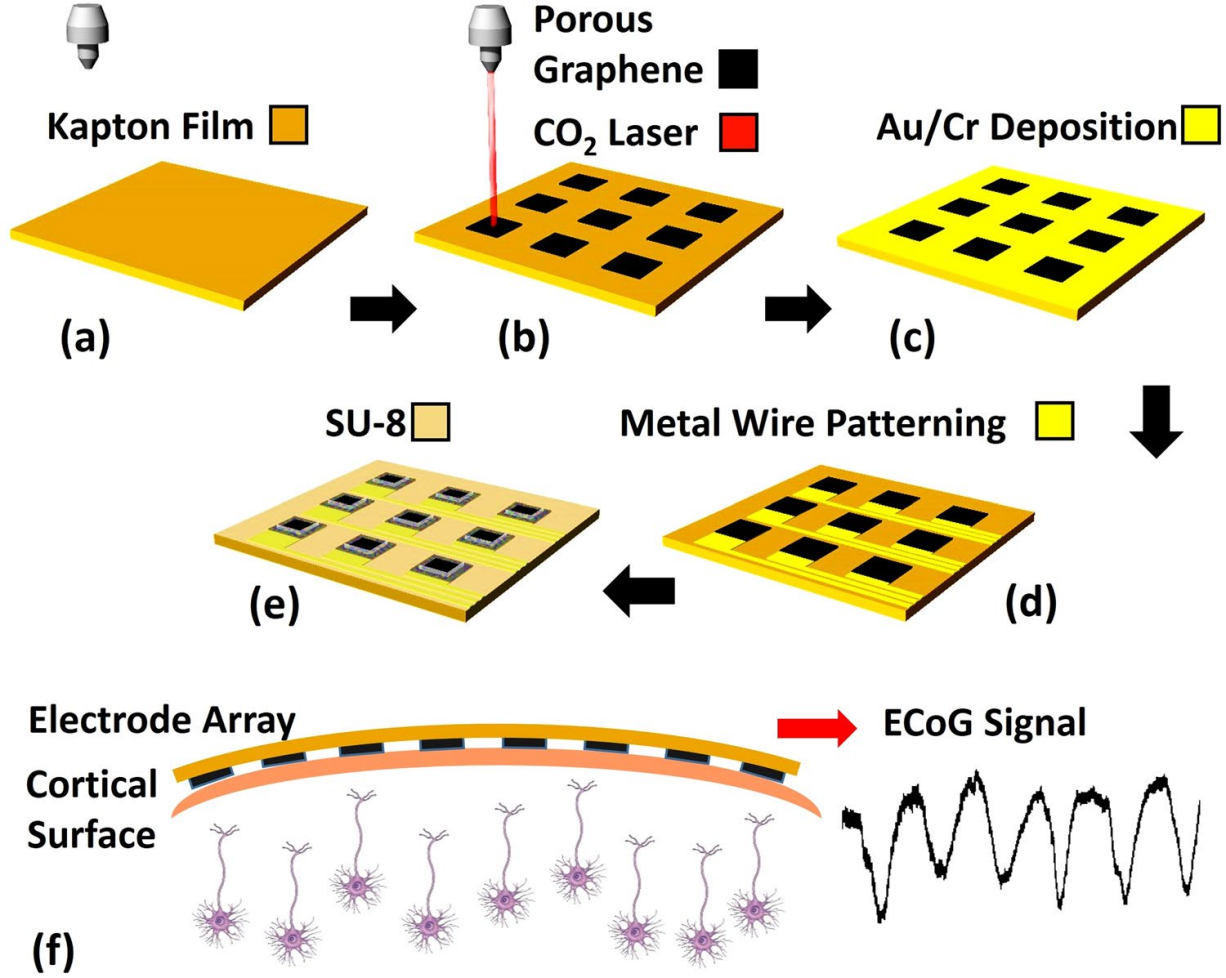
Fabrication process of the porous graphene electrode array. (**a**) A 50 μm thick Kapton FPC film is used as substrate. (**b**) Porous graphene is fabricated with laser pyrolysis. (**c**) 10 nm Cr and 100 nm Au are deposited with E-beam evaporator. (**d**) Metal leads were patterned with photo-lithography and wet-etching. (**e**) 8 μm thick SU-8 is defined with photo-lithography as the encapsulation layer. (**f**) A schematic showing the fabricated porous graphene electrode array placed on top of the cortical surface during the recording.

**Figure 2:**
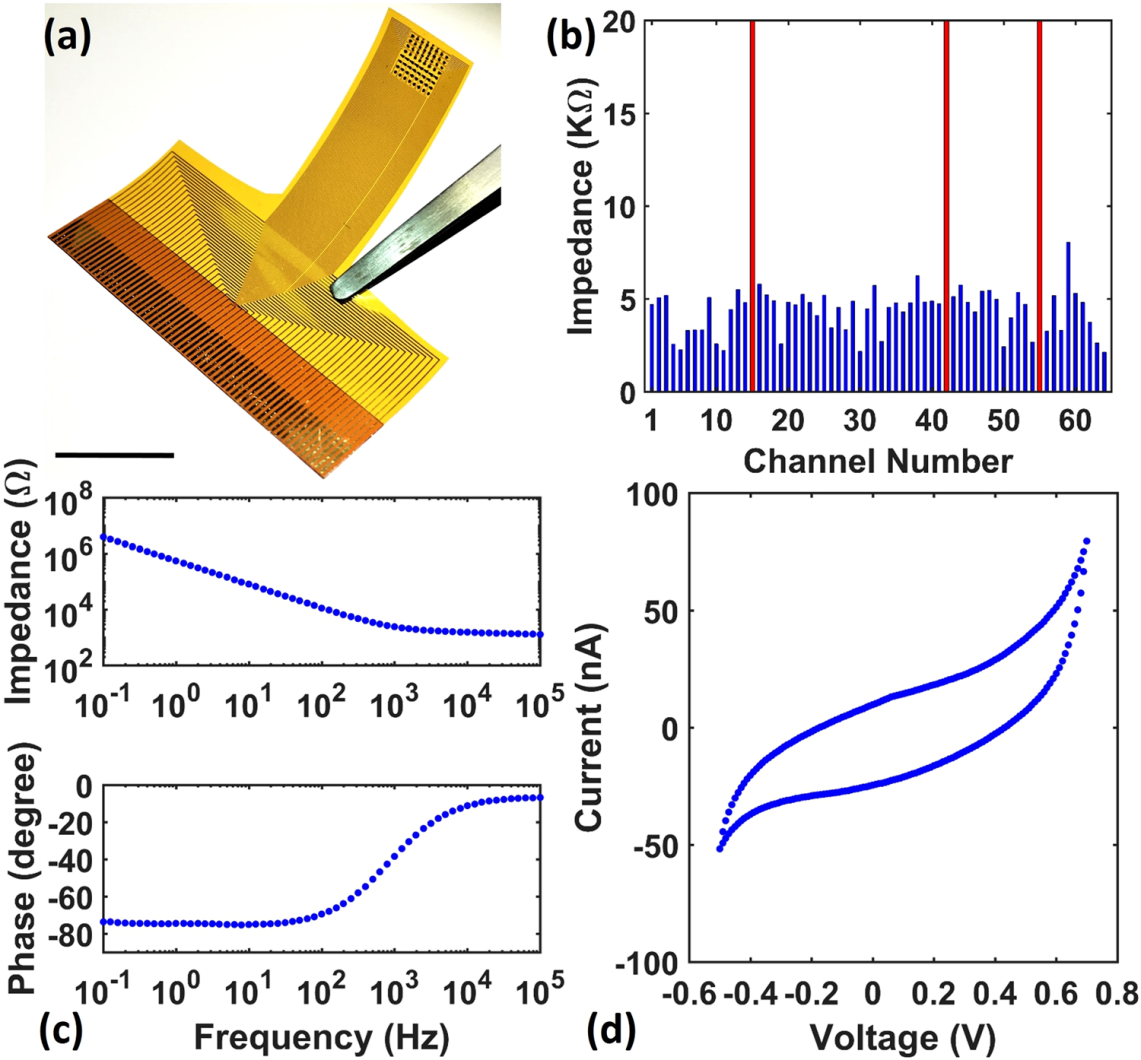
Picture of array and electrochemical characterization. (**a**) A picture of the 64 channel porous graphene electrodes array. The scale bar: 1 cm. (**b**) The impedances of all the 64 channels of the porous graphene electrode array. The three high impedance channels are marked as red (>2 MΩ). (**c**) EIS measurement and the (**d**) CV result of one representative channel.

Porous graphene microelectrode arrays were fabricated using the process shown in Fig. 1. The porous graphene was formed by pyrolysis using a carbon dioxide laser with a wavelength of 10.6 μm on polyimide film (Kapton), as shown in Fig. 1a,b. 10 nm chromium and 100 nm gold were deposited using E-beam evaporation (Fig. 1c). The metal wires and contact pads were then patterned with photo-lithography and wet-etching (Fig. 1d). Finally, 8 μm thick SU-8 was used as the encapsulation layer and patterned with photo-lithography, leaving the active electrode areas exposed (Fig. 1e). The electrode openings are 250 μm × 250 μm. The spacing between adjacent openings is 500 μm. Figure 2a shows a picture of the fabricated porous graphene electrode array. Figure 2b shows the impedances of all the 64 channels of the porous graphene array measured by the Intan RHD2000 evaluation system at 1 kHz. The average impedance is ~4.36 kΩ. The cyclic voltammetry (CV) and the electrochemical impedance spectroscopy (EIS) results of one representative channel are shown in Fig. 2c,d.

We performed *in vivo* animal experiments on three adult rat models. The porous graphene electrode array covered a large area of the rat dorsal cortex, including part of the motor cortex and sensory cortex. The picrotoxin was applied topically to induce epileptic activity. We used two Intan RHD2132 electrophysiology chips and an Intan RHD2000 evaluation system to record the μECoG data. During the experiments, we focused on two different neural activity states, namely the theta oscillations and the epileptic activity induced by the picrotoxin.

**Figure 3:**
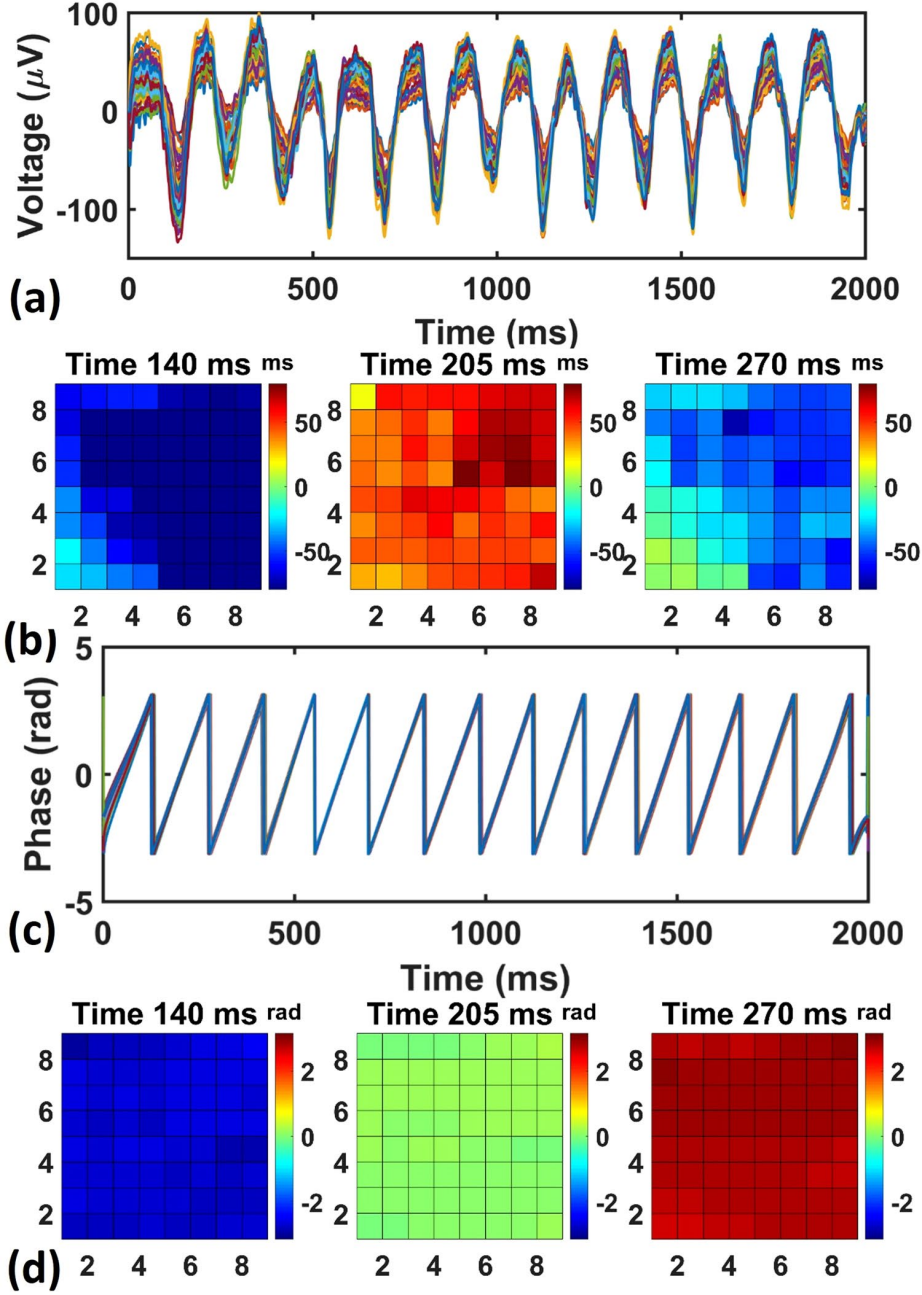
Spatiotemporal analysis of theta oscillations. (**a**) Raw data recorded in all the channels, showing global 7 Hz low-amplitude theta oscillations under the anesthesia. (**b**) The amplitude maps for all the 64 channels at three different time points drawn from (**a**). (**c**) The phases of the electrical recordings that are filtered at theta band (5 Hz–9 Hz). The traces from all the 64 channels are plotted together to show synchrony among all the channels. (**d**) The phase maps at three different time points showing global stationary waves.

Figure 3a shows a representation of the raw data obtained from recording low-amplitude 7 Hz theta oscillations observed across all the 64 channels of the electrodes array. Figure 3b shows amplitude maps taken at three different time points from Fig. 3a. The amplitudes of the theta waves show slight differences across various channels. In addition to the amplitude, we also computed the phases of these theta oscillations recorded in the 64 channels (Fig. 3c). The phases of the theta oscillations overlap closely with each other. This can also be seen from the phase maps at specific time points (Fig. 3d). An example video showing the recorded theta oscillation is available in the supplementary materials (Supplementary Material Movie 1). These results suggest that the recorded theta oscillations were mainly global stationary waves that did not propagate across the cortex surface. The recorded electrical signals in all the channels across the array rose and fell synchronously, which implies that theta waves were generated by a global mechanism, synchronizing most of the cortex simultaneously. Note that, in literature, theta oscillations were commonly observed under Ketamine anesthesia in EEG studies^12,13^. Supplementary Fig. 1 shows the raw recording data and the spectrogram of the signals recorded by one representative channel of the porous graphene array, showing obvious oscillations in the theta frequency band. Besides the theta oscillations, we also recorded 1–2 Hz slow oscillations as shown in the supplementary materials (Supplementary Fig. 2). Different from the theta oscillations, we found that the slow oscillations were not stationary and could travel across the recording area in various directions with different speeds (Supplementary Fig. 3), which was consistent with previous studies on traveling slow oscillations^14–16^.

Next, we investigated the spatial-temporal behavior of cortical potentials during epileptic activity. The neural recording data was preprocessed as follows: First, for the epileptic recordings, we low-pass filtered the data at 120 Hz, which preserved the shape of epileptic waveforms and also reduced the high frequency noise. In order to analyze the spatiotemporal characteristics of each epileptic spike event, a voltage threshold was applied on the recordings to extract the time series within a certain time window when a large epileptic spike occurred. Since the ictal spikes and inter-ictal spikes had different temporal widths in the electrical recordings, the window size was chosen to be 100 ms for ictal spikes and 500 ms otherwise to make sure there was only one epileptic spike in each time window. Note that the term “epileptic spikes” used here refers to the high amplitude ictal or inter-ictal waveforms observed in the ECoG recordings, not the action potentials or single unit activities associated with single cell firings.

**Figure 4:**
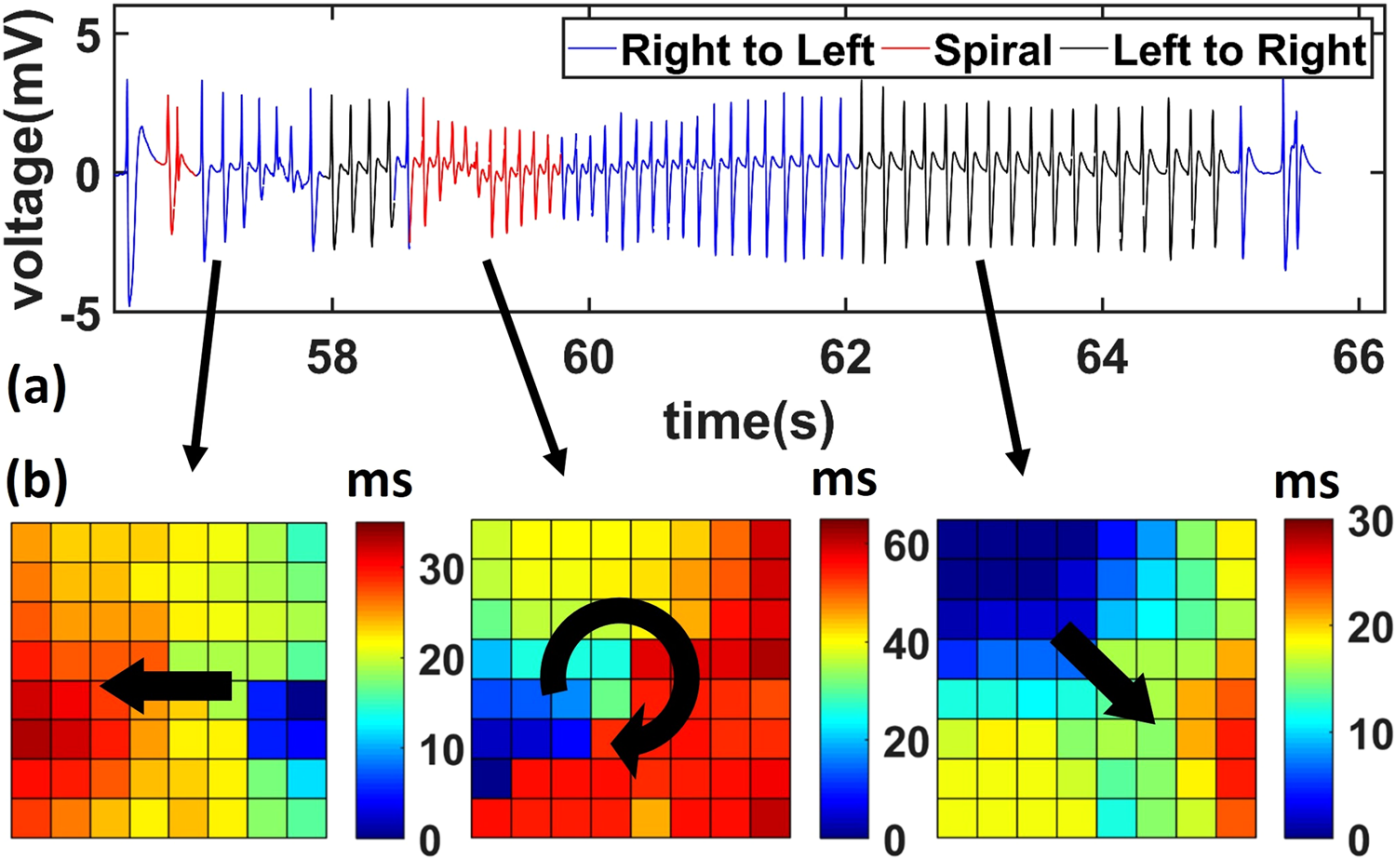
Spatiotemporal analysis of seizure. (**a**) Representative raw electrical recording data from one channel during the seizure. (**b**) Representative delay maps showing various types of propagations (Left: right-to-left wave; Middle: spiral wave; Right: left-to-right wave) corresponding to different epileptic spikes during the seizure shown above.

On the contrary to the theta oscillations, the epileptic spikes were less synchronized and exhibited various types of propagation patterns. The typical raw neural recordings from one of the channels during a seizure are shown in Fig. 4a. Unlike the small amplitude theta oscillations, the epileptic activity has amplitudes of several millivolts. The different propagation patterns are marked by different colors. The right-to-left propagations are marked in blue, whereas left-to-right and spiral waves are labeled in black and red respectively. During the seizure, different propagation types actively switched between each other. Example videos showing the traveling waves can be found in the supplementary materials (Supplementary Movies 2 and 3). To better quantify the propagation characteristics of right-to-left, left-to-right, and spiral waves, three typical time delay maps are shown in Fig. 4b, each corresponding to one type of epileptic propagation events indicated by the black arrows. The color blocks encode the time delays between a specific channel and the reference channel. The right-to-left wave started from the right middle edge of the array and propagates gradually to the left edge. The spiral wave initiated from the lower left edge and traveled clockwise through the entire plane. The left-to-right wave started from the top-left corner and propagated diagonally to the bottom right corner. Also, during the inter-ictal stages, most of the propagating waves were right-to-left, whereas the spiral and left-to-right waves occurred predominantly during ictal stages. These results indicate that the brain was less globally synchronized during the epileptic stages as opposed to the theta oscillations. Strong and locally synchronized neural activities initiated in one region, which gave rise to the high amplitude ECoG signals in the nearest electrodes. Then, the activation quickly spread to nearby brain regions within a time delay of tens of milliseconds. For each epileptic spike, the propagating waves could have taken different paths, which might have resulted from different activation orders of various regions. Since the spirals required longer activation paths than simple left-to-right and right-to-left waves, it also shows longer time delay. The spirals are hypothesized to spatially coordinate different parts of the brain and have been found in normal neocortical activities through voltage-sensitive dye imaging^17,18^ and macroscopic scale ECoG recording^2^, as well as in pathological epileptic networks through microscopic scale ECoG studies^4,5^.

**Figure 5:**
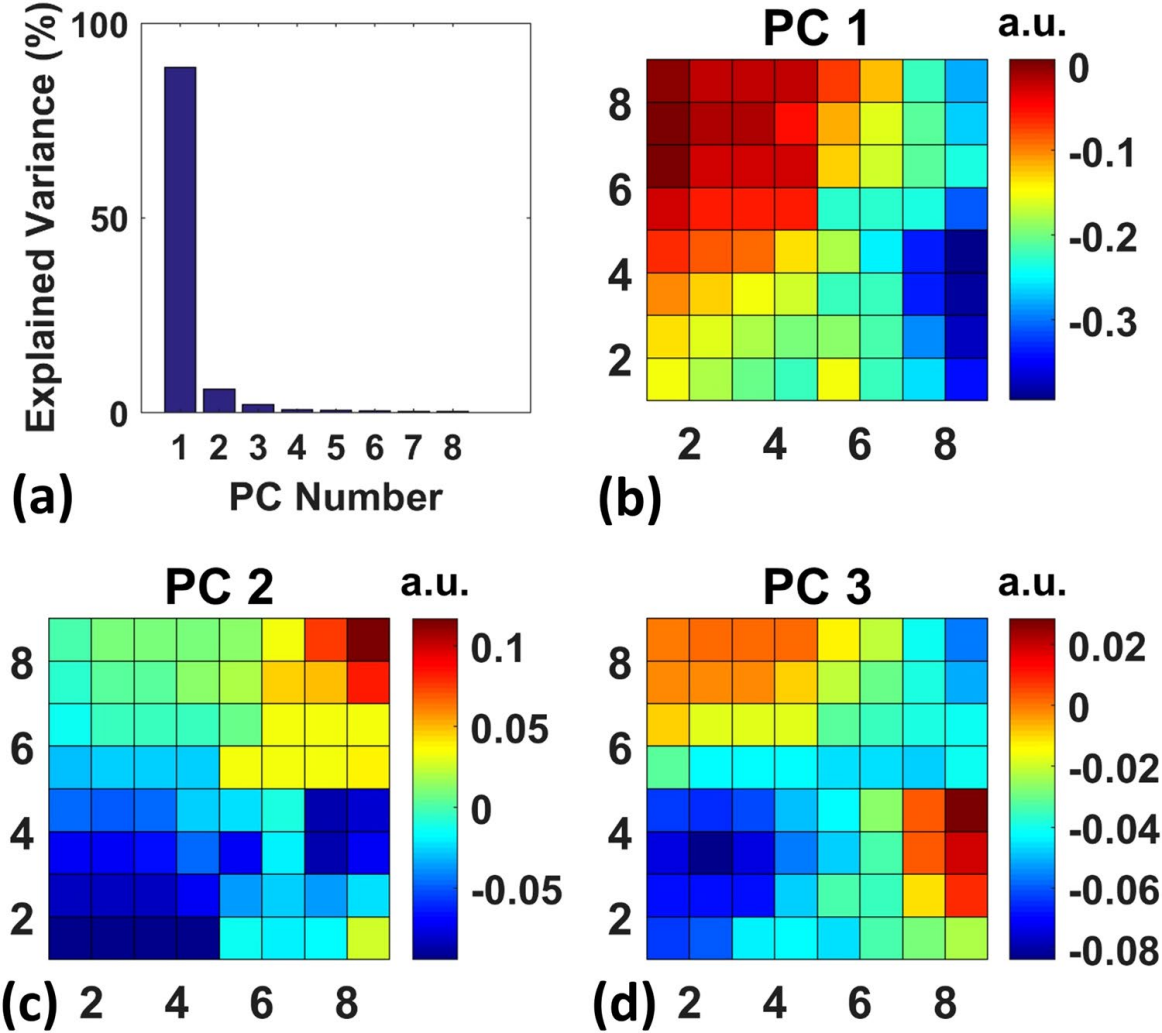
Principal component analysis (PCA) results of the epileptiform waves. (**a**) The principal component analysis result shows that 92% of the variance can be explained by the first 3 principal components. (**b**,**c**) and (**d**) show the coefficients of the first three principal components from the PCA analysis results, which implies different propagation directions of the recorded potentials.

Based on the observations that different propagating waves existed during the seizures, we further investigated the propagation patterns during epileptic activities. Specifically, we classified the epileptic spikes based on the algorithm explained in detail below. Since the aim was to classify different epileptic spiking events according to their propagation directions, we inspected the time delay between different channels. Thus, for each specific epileptic spike, we chose the time delay between different channels as features. In order to do that within each time window, we computed the cross-correlation coefficient between the signals from different channels. The time delay was then determined as the temporal shift that maximized the calculated correlation value. In order to get the features for each epileptic spike, one way was to repeat this process for all the pairs of the 64 channels. In that case, for each epileptic spike, we could obtain a 2D time delay matrix of size 64 by 64, which needed to be flattened into a 1 by 4096 vector for the later clustering algorithm. However, even though this time delay matrix itself was only two dimensional, the underlying feature space represented by the 2D delay matrix consisted of many pairwise time delays, each contributing one dimension. Therefore, the feature space was actually very high dimensional and would degrade the performance of the clustering algorithm due to the curse of dimensionality, a phenomenon where a specific machine learning algorithm succeeds with low dimensional data, but fails with high dimensional data^19^. Also, this 4096 dimensional feature space contained redundant information due to the antisymmetric nature of the delay matrix. Therefore, to reduce the dimensionality, we only took the first column (a 64 by 1 vector) of the time delay matrix as features, which contained the pairwise time delays between all the channels and the first channel. Note that by doing this operation, the sufficient information to classify the epileptic spikes is still preserved, since the first column of the 64 × 64 delay matrix already exhibits sufficient amount of differences for distinct types of propagating epileptic spikes. Then, similar with previous studies that investigated the propagating waves based on high dimensional time delay feature space^4,5^, we employed principal component analysis (PCA) on the 64 dimensional data sets. Figure 5a shows that the first 3 principal components are sufficient to explain ~96.44% of the variance in the data. Figure 5b–d illustrates the coefficients of the first three principal components organized in an 8 by 8 matrix. These results imply most propagation patterns are directional waves. To classify the epileptic waves, we projected each data sample to the 3 dimensional space spanned by the first 3 principal components and applied a k-means clustering algorithm. To choose a proper cluster number, we used heuristics by first computing the within cluster sum of squares W_k_ for different cluster numbers k, and then choosing the cluster number that gives the “elbow” point, which is defined to be the location where the decrease of W_k_ is significantly flattened. This “elbow” method is easy to implement and commonly used in the cluster analysis literature^20^. We also inspected the data from representative recording segments for tentative cluster numbers. Based on these observations, the cluster number was chosen to be 3. The clustering result showed that, among the 1615 epileptic spikes recorded from 3 rats, ~92 percent of them were traveling from right to left, while the rest of them were left-to-right and spirals waves with almost equal occurrence percentages. Note that different studies have reported different numbers and ratios of the propagating waves observed during epilepsy^4,5^. Also, there are inter-subject differences in terms of the number and spatiotemporal characteristics of different propagation types. We anticipate that specific conditions used for epileptic drug delivery methods used in these studies might affect types and directions of the observed propagation patterns.

From the above spatial-temporal analysis results, we have seen that the cortical potentials actively switch among distinct propagating patterns during the seizure. Since the ECoG recordings reflect the overall activities of the underlying neural circuits, the above observations lead to the question whether the network state changes in the neural circuits are indicated by these different propagation patterns. Therefore, we employed network analysis methods that were commonly used to study the functional connectivity of complex brain systems^9,21,22^ to investigate the functional connectivity of the cortical neural circuits. Then, we compared the network states with the propagating wave patterns.

Before running the network analysis, we preprocessed the data by subtracting the signals in all of the channels by their mean to remove the common mode noise. Then we treated each electrode channel as a node that reflected the overall activity of the local neural populations directly beneath it. Similar with the spatial-temporal analysis described above, we segmented the recordings at each epileptic spiking event during the seizure and evaluated the functional network connectivity for each segment. Since the electrode dimensions were small and we were interested in analyzing the functional connectivity based on local neural activities, it was important to remove the low frequency background signals that were present in all the 64 recording channels. Typically, these large-amplitude low-frequency components are considered as a consequence of volume conduction^23^ and may bias the functional connectivity analysis into the positive direction, which increases the false positive rate. Therefore, to reduce the possible undesirable effects of volume conduction, we band-pass filtered the data in the high-gamma range (80–120 Hz), which has been reported to closely correlate with the overall firing rates of the local neural populations^24,25^. To evaluate the connection strengths between the nodes, we adopted the cross-correlation method to measure the functional connections between the neural populations under different channels. Note that cross-correlation connectivity measurement is a power-based connectivity evaluation method, which is robust to different time delays. It examines the covariance of the recorded signals in designated frequency bands within a certain time delay range and doesn’t assume instantaneous responses in different functionally connected channels. This is a suitable method in our case, since we want to compare the network states with different propagation patterns. A connectivity measure that is sensitive to time delay will probably bias the comparison. Thus, for each recording segment, we obtained a cross-correlation based connection matrix. Similar to another epileptic network study^9^, we employed the Pearson correlation to quantify the similarity between any connection matrix pairs. Finally, we got an undirected weighted graph described by a similarity matrix whose elements represent the similarities between different network connectivity patterns during the spiking events (Supplementary Fig. 4). Based on this similarity matrix, we employed the community detection algorithm, which maximized a modularity quality function Q^26^ by a Louvain-like greedy algorithm^27^.

**Figure 6:**
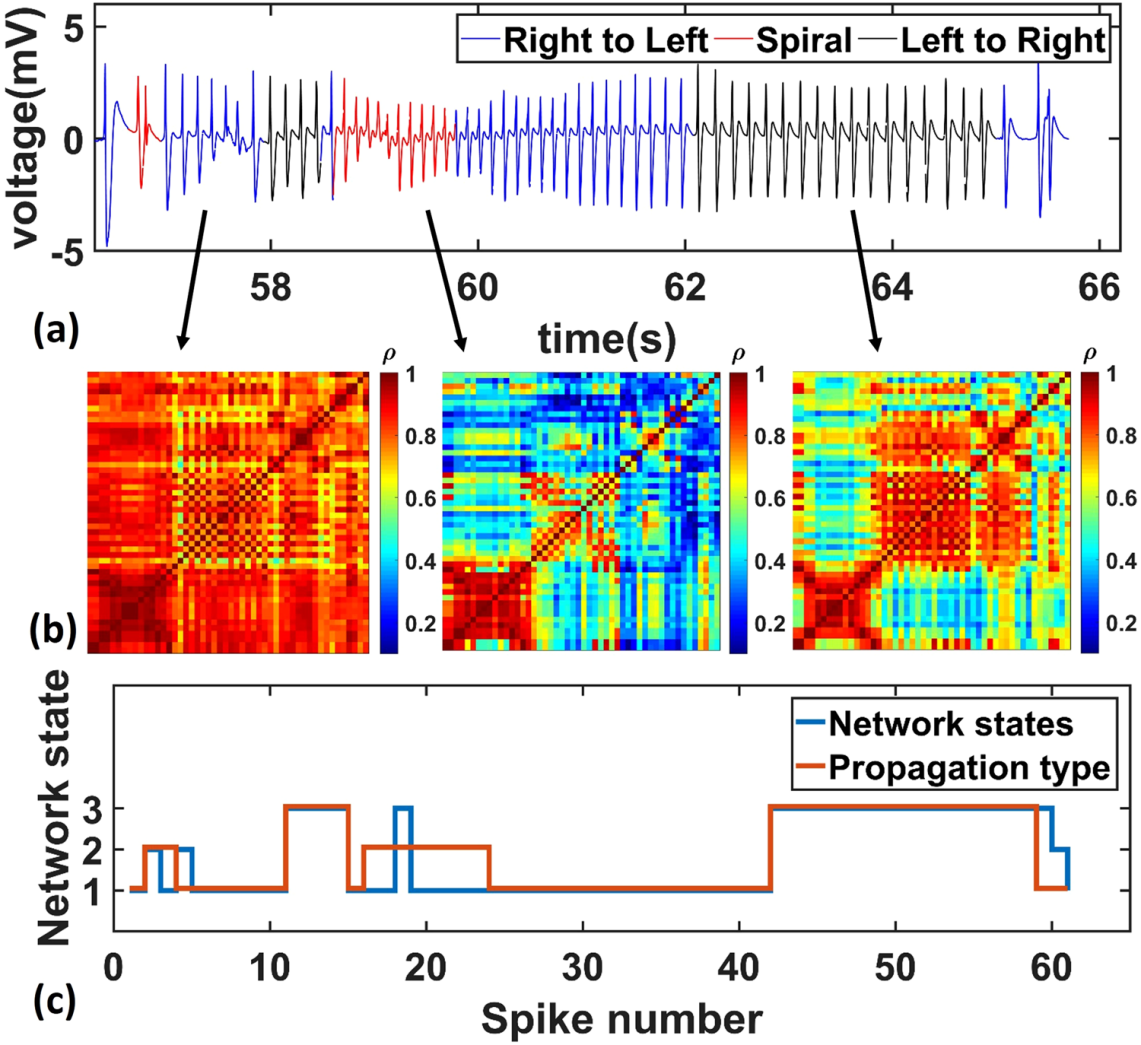
Comparison between propagation patterns and the network states during one seizure. (**a**) The same electrical recording as Fig. 4a. (**b**) Connection matrix for different propagation patterns, such as spirals (left panel), right-to-left wave (middle panel), and left-to-right wave (right panel). (**c**) A comparison between propagation types and network clustering results showing consistency between the two.

The network clustering result for a representative seizure is shown in Fig. 6. Figure 6a shows the electrical recordings of the seizure where the red color indicates the spiral waves and the blue and black colors represent the right-to-left and left-to-right propagations respectively. The typical connection matrices corresponding to three different propagating waves are shown in Fig. 6b. The strong connections are indicated by red, whereas the weak connections are marked in blue. It can be seen that distinct network configurations evolve actively along with the different propagating waves during the seizure. Also, as shown in Fig. 6c, the clustering result of the network states mostly coincides with the propagation types of the epileptic spikes. In another seizure recording, the network changes are also largely consistent with the evolution of the propagating patterns (Supplementary Material Figs 5 and 6). To inspect whether the changes in network configurations are correlated with the changes of cortical propagating waves, we performed the chi-squared test on the clustered network states and propagating types for all the 1615 epileptic spikes we recorded from 3 rats. Because the clustering result of the propagating types could be different due to random seeds, we ran the k-means clustering algorithm and the chi-squared test 100,000 times. The resulting χ^2^ statistics are shown in the histogram in Supplementary Fig. 7a. In all the runs, we consistently rejected the null hypothesis that the network states are independent of the propagation types (chi-squared test, df = 4, p-values < 0.0001). To further quantify the strength of association between the network states and the propagation types, we computed the Cramer’s V using the 100,000 chi-squared statistics obtained above. The result in Supplementary Fig. 7b shows that the correlation between the network states and the propagating types is significantly higher than chance (rank sum test, n = 100,000, p-value ≪ 0.0001). Overall, these results indicate that the changes in network configurations are significantly correlated with the propagation patterns of the epileptic spikes.

## Discussion

In this work we employed high-density porous graphene arrays for recording cortical traveling waves. Different from the porous graphene arrays presented here, monolayer graphene, a two-dimensional single-atom thick material, has also been investigated for multimodal neural interfaces in our previous studies^28,29^. Owing to the high optical transparency and the absence of light-induced artifacts, monolayer graphene electrodes enable successful combination of electrophysiology with optical imaging techniques such as 2-photon microscopy^29,30^ or optogenetic stimulation^11,29^ in multimodal experiments. However, monolayer graphene electrodes exhibit relatively high electrochemical impedance due to their planar surface and small number of active sites for charge transfer. On the other hand, the porous graphene has a 3D structure consisting of flakes of multilayer graphene and graphene oxide^31^. This 3D structure significantly increases the effective surface area and number of active sites, leading to extremely low impedance and hence high signal-to-noise ratio (SNR) in electrical recordings. Porous graphene can simply be fabricated on polymer substrates using laser pyrolysis, which is easily scalable to large areas. In addition, the porous graphene microelectrodes have a high charge injection capacity (3.1 mC/cm^2^) that is particularly suitable for cortical microstimulation^11^, different from monolayer graphene, which has a limited charge injection capacity. Because of the key advantages of high density across large areas and high SNR in recordings, porous graphene microelectrode arrays were used to record traveling cortical waves with high fidelity in this work.

By combining the high fidelity neural sensing and effective neural stimulation enabled by the low impedance and high charge injection capacity respectively, our porous graphene array technology can be applied in both basic neuroscience research and clinical studies of neurological disorders. Recently, it has been shown that closed-loop electrical stimulations can effectively control epilepsy^32^ and achieve tremor suppression^33^. Porous graphene array technology can be adopted to achieve high-resolution microstimulation for precise targeting of brain tissue in closed-loop neuromodulation applications. Finally, low impedance of the porous graphene electrodes can allow scaling down electrode dimensions and increasing electrode density to investigate epileptic activity across multiple brain circuits with very high resolution towards precise detection of seizure onset zones in the brain.

Besides the pathological epileptic waves reported here, physiological traveling waves have also been reported by various studies for different species under distinct cognitive states. These traveling waves exist in both macroscopic and mesoscopic levels^1^. For example, people have observed brain-wide traveling slow oscillations during Non-REM sleep in human EEG studies^34^. In ECoG recordings, the phase of the traveling alpha waves was reported to modulate the gamma band power in humans during awake state^35^. In recent years, it was shown that the spindles observed commonly during human sleep were mostly rotating waves that traveled across different brain regions^2^. It was hypothesized that these widespread traveling waves integrated the information across different brain regions and played an important role in learning and memory. Besides the brain-wide propagations, the traveling waves have also been found in local cortical networks. In a study of the primary visual cortex in awake monkeys, it was shown that traveling waves induced by the visual stimuli could span a few millimeters in V1^36^. Therefore, recording and studying traveling waves have been suggested to be critical for understanding the information processing within and across cortical networks. To accomplish this goal, the spatial resolution of the electrode arrays and the signal-to-noise ratio of the electrical recordings are very important. Besides the pathological epileptic traveling waves presented here, our high-density low impedance porous graphene array can also be used in studying the spatiotemporal dynamics of physiological waves to investigate their roles in different cortical networks.

## Conclusions

In this work, we demonstrated that the high-density porous graphene electrode arrays provide high spatiotemporal resolution to capture the rich dynamics of the propagating cortical waves. The flexibility of the array and the low impedance of porous graphene electrodes enabled the recording of cortical potentials with high fidelity. *In vivo* experiments in rats revealed that low amplitude theta oscillations were globally stationary across large areas with phase synchronized tightly in different electrodes of the porous graphene array. The epileptiform activity on the other hand was not stationary and exhibited various types of spatiotemporal propagating patterns along the cortical surface, including right-to-left, left-to-right, and spiral waves, which were less synchronized than theta oscillations. Finally, the network analysis on the local neural population activity suggested that during the ictal stage, the neural circuits underwent various functional connectivity changes, which were consistent with the evolution of different propagating waves recorded from the cortical surface.

## Methods

### Fabrication process of the porous graphene electrode array

First, we used a laser engraving and cutting system (PLS6.75, Universal Laser Systems Inc.) equipped with a 10.6 μm wavelength CO_2_ laser to irradiate polyimide films (50 μm thick, Kapton). After the patterning, the polyimide was first rinsed with acetone, isopropyl alcohol, and deionized water for cleaning, and later de-moisturized with a hotplate at 150 °C for 5 minutes. In order to keep the polyimide film flat during the whole process, it was attached to a 4 inch Si wafer spin-coated with polydimethylsiloxane (PDMS). Then, we deposited 10 nm Cr and 100 nm Au with electron-beam evaporation. The metal wires and contact pads were patterned with S1818 photoresist and wet etching. A 9 μm thick SU8-2007 was spin-coated and patterned to serve as the encapsulation layer. The dimension of each electrode is 250 μm × 250 μm and the distance between adjacent electrodes is 500 μm.

### Electrochemical characterization

We set up the Gamry Reference 600 potentiostat in the standard three-electrode configuration in 0.01 M PBS solution. Ag/AgCl and Pt were chosen to be the noncurrent-carrying reference electrode and counter electrode respectively. The CV measurements were done by sweeping the potential of the working electrode within the water window at a rate of 100 mV/s. The EIS measurements were performed between 0.1 Hz and 300 kHz using 10 mV RMS AC voltage.

### *In vivo* neural recording

Experiment procedures were approved by the Institutional Care and Use Committee of the University of Pennsylvania. *In vivo* data presented in the paper (Figs 3–6) were from representative recordings collected in three different acute experiments, each of them lasting for 5–6 h. In animal experiments, we used a ketamine (60 mg/kg), dexdomitor (0.25 mg/kg) solution to anesthetize the rats. In these experiments, ketamine-based anesthesia is preferred over isoflurane, since isoflurane is known to suppress epileptic activity^37^. Part of the skull was removed to expose a large area of the cortex. The reference electrode was connected to a screw placed in the backside of the rat skull. The porous graphene microelectrode array was placed on the exposed cortical surface. The electrophysiology data were recorded at 10 kHz per channel using two Intan RHD2132 electrophysiology chips and an Intan RHD2000 evaluation system. In these experiments, we ensured that breathing artifacts are eliminated by proper placement of reference electrode and wires to avoid any electrical or mechanical coupling with the animal body.

### Spatiotemporal data analysis

All the data processing procedures were done in MATLAB. In order to investigate spatiotemporal characteristics of traveling waves in Fig. 4, unfiltered raw data recorded by 64-electrode porous graphene array were used. Filtering to high gamma range was only applied to network analysis, which will be discussed in the next paragraph. To extract the phase of the theta oscillations, we first applied a 4^th^ order Butterworth band-pass filter (5 Hz–9 Hz) to the raw signals recorded by all the 64 channels. Then we used Hilbert transform to get the instantaneous phases of the theta oscillations. For the epileptiform activities, to increase the signal-to-noise ratio, a 6^th^ order Butterworth low-pass filter (120 Hz) was applied to every channels to remove the high frequency noise. Then we employed a voltage threshold of 500 μV to extract the epileptic spikes during the entire recording. The time window was 500 ms for inter-ictal spikes and 100 ms for ictal spikes to make sure each time window only has one epileptic spike. To determine the time delay between the recording channels, we first computed the cross-correlation between different channel pairs and then choose the time lag that maximized the correlation value. Note that, the cross-correlation has been used as a common method to obtain the time delay between different channels in both *in vivo*^15,16^ and *in vitro* studies^14,38^ of traveling slow oscillations. Alternatively, directly computing the time lag between the peak values in the recordings from two channels gave similar results.

### Network analysis

Each electrode was treated as one node and the connection strengths between different nodes were measured by the cross-correlation values. Thus, for each time segment, we obtained an undirected weighted graph that is represented by a connection matrix whose columns and rows stand for different electrodes. To determine the similarity between two network connections, we computed the Pearson correlation between any two connection matrices. Thus we obtained a similarity matrix that has N columns and N rows where N is the number of time segments we wanted to evaluate. In other words, we had another undirected weighted graph whose nodes stood for different time segments and edges represented the similarity between the network connections at different time points. Based on this graph, we used a MATLAB toolbox which efficiently implemented the community detection algorithm that clustered the most closely connected nodes into the same communities.

## Electronic supplementary material

Supplementary Material

Supplementary Movie 1

Supplementary Movie 2

Supplementary Movie 3

## Data Availability

The ECoG data and the custom MATLAB code are available upon reasonable request.
