# Supplementary material for: High-Density Porous Graphene Arrays Enable Detection and Analysis of Propagating Cortical Waves and Spirals
Source: Sci Rep. 2018 Nov 20;8:17089. doi: 10.1038/s41598-018-35613-y (PMC6244298; doi:10.1038/s41598-018-35613-y)
Supplement: Supplementary file 1 — Supplementary Material [file 41598_2018_35613_MOESM1_ESM.pdf]

Supplementary Material

High Density Porous Graphene Arrays Enable Detection and Analysis of Propagating Cortical Waves and Spirals

Xin Liu[1], Yichen Lu[1], and Duygu Kuzum[1]

[1] Department of Electrical & Computer Engineering, University of California, San Diego, La Jolla, CA 92093, USA

E-mail: dkuzum@eng.ucsd.edu

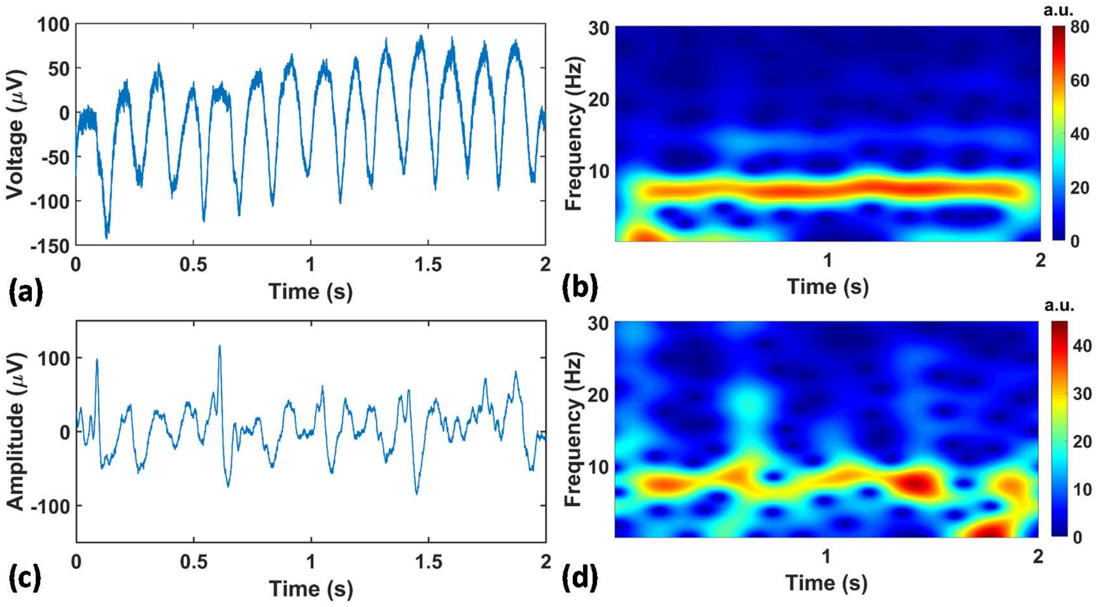

Supplementary Figure 1. (a) The same raw data shown in Figure 3a in the main text. (b) The spectrogram showing theta oscillation for data in (a). (c) Raw recorded signals by microwire electrodes under the same experimental settings. (d) The spectrogram for data shown in (c). Note the dominant power in the theta bands.

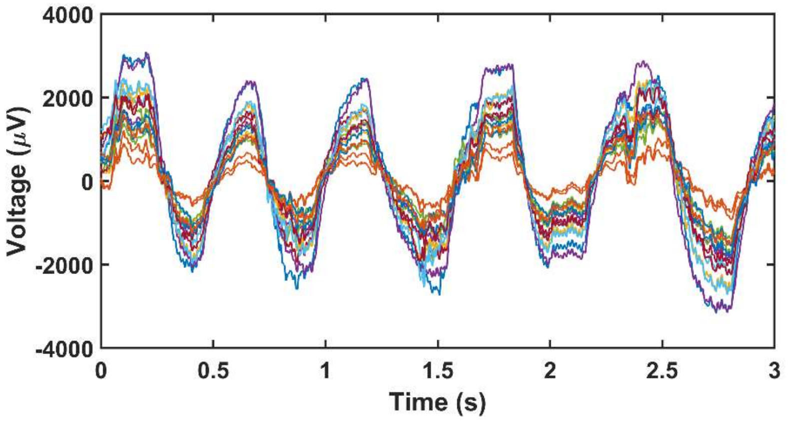

Supplementary Figure 2. Example ECoG recordings showing the 1-2Hz slow oscillations.

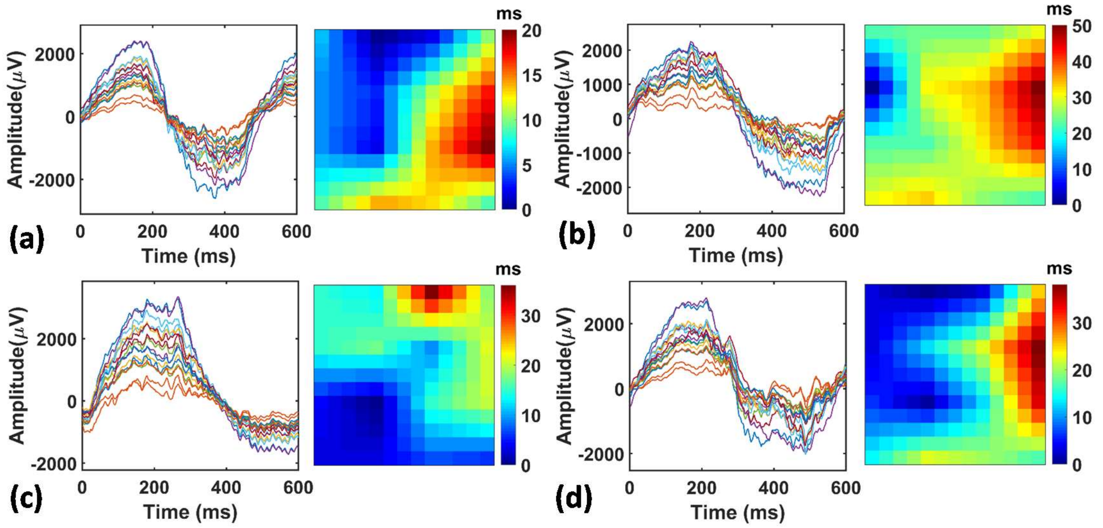

Supplementary Figure 3. Representative propagations for the observed slow wave oscillations. The electrical signals shown on the left are raw data without any filtering. The color maps shown on the right are the delay maps obtained using interpolation. The traveling speeds are (a) 106 mm/s, (b) 30 mm/s, (c) 32mm/s, (d), 28 mm/s

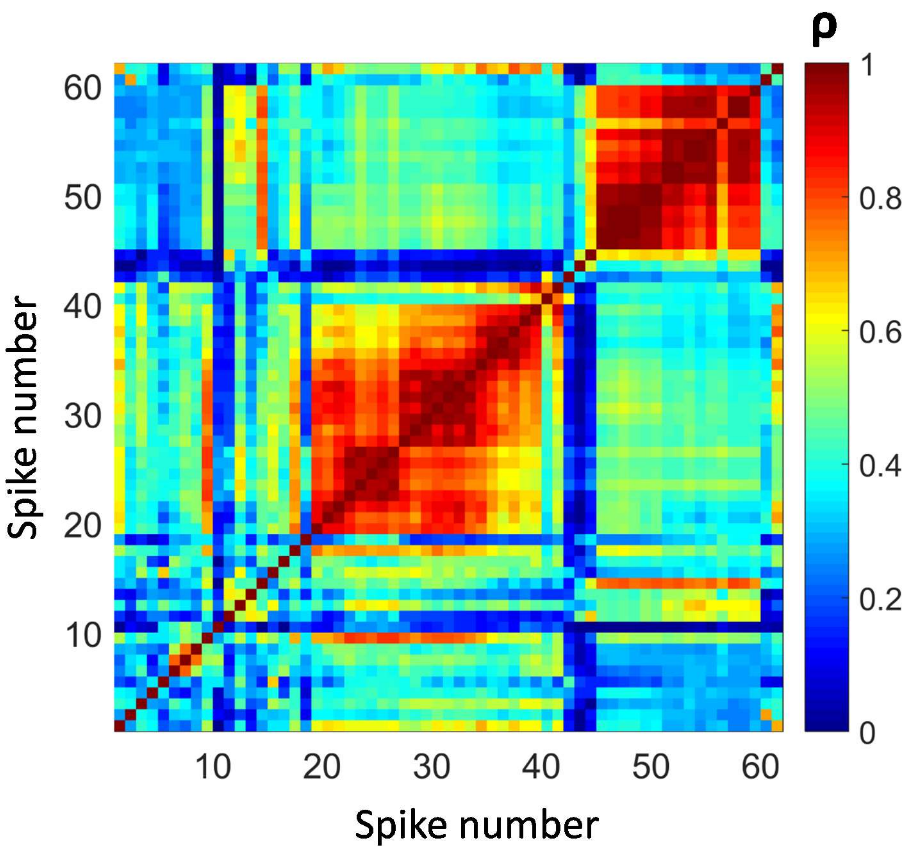

Supplementary Figure 4. Similarity matrix for the seizure presented in Figure 6 in the main text. The red color indicates high similarity between the functional network connections of two spiking events. The blue color represents low similarity.

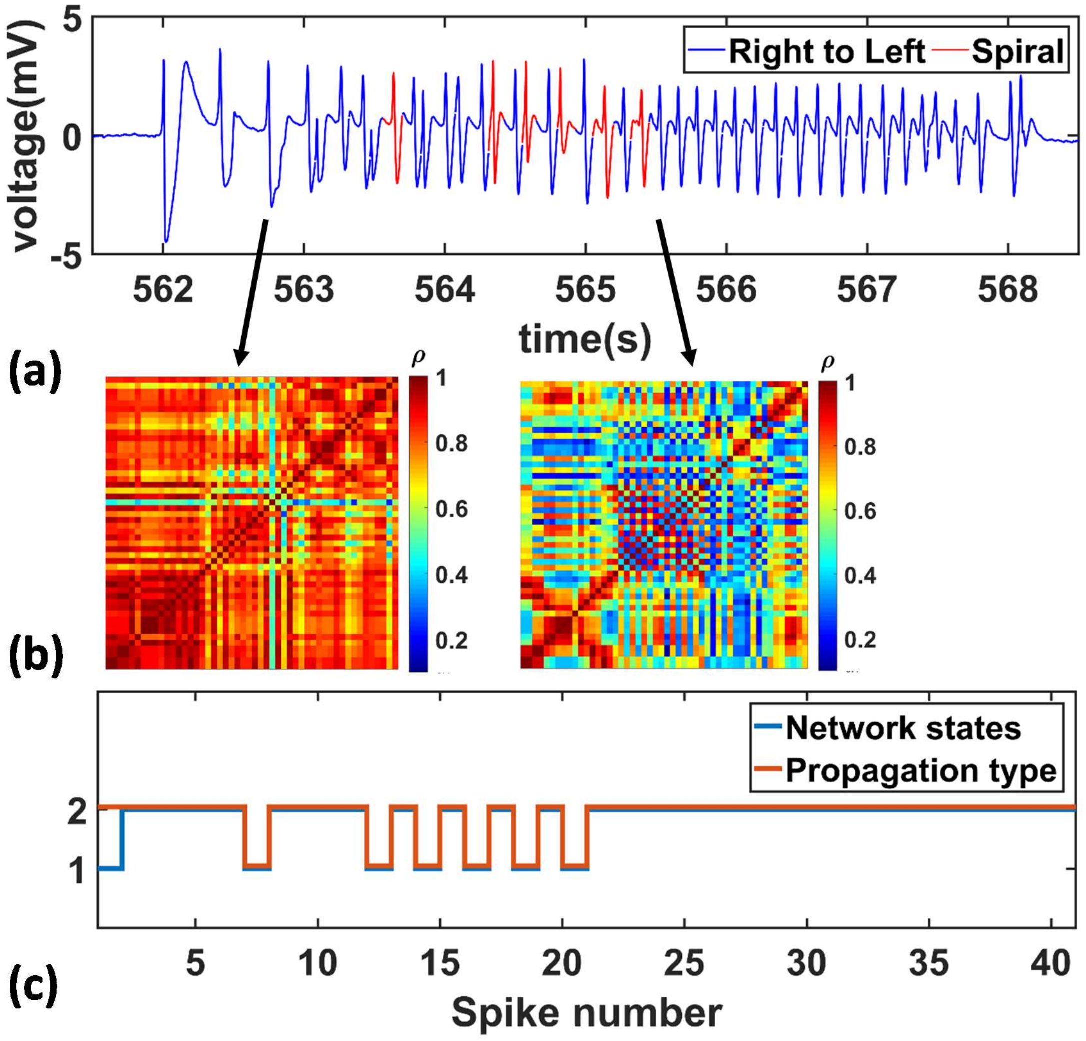

Supplementary Figure 5. Comparison between propagation patterns and the network states during another seizure. (a) The electrical recording from one channel. The red color represents spiral waves and the blue color stands for right-to-left waves. (b) Representative connection matrix for different propagation patterns, such as right-to-left wave (left panel), and spirals (right panel) (c) A comparison between propagation types and network clustering results showing consistency between the two

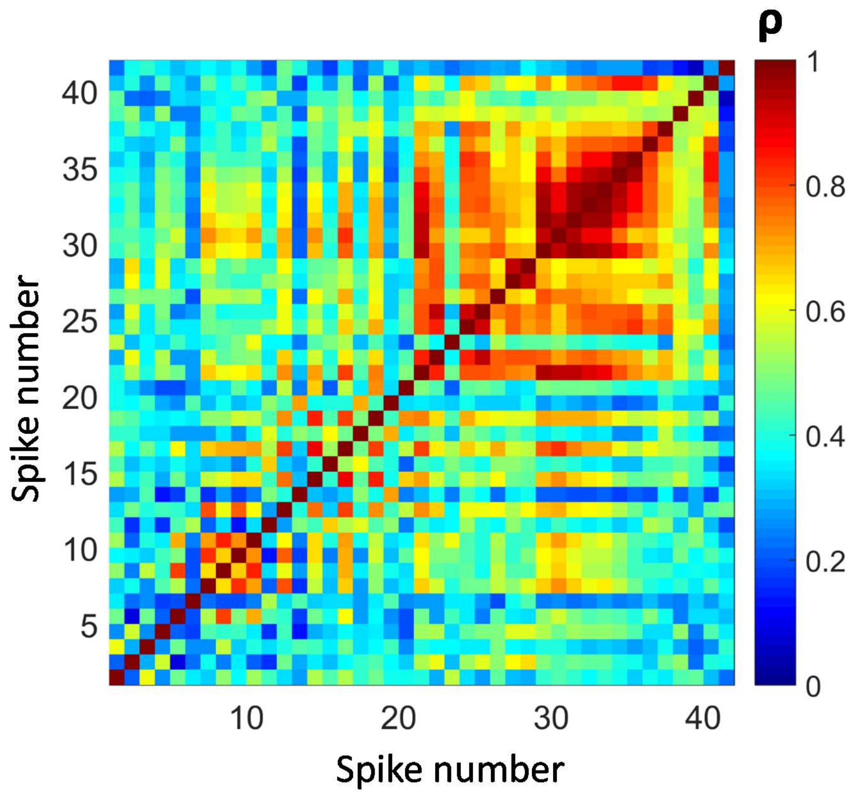

Supplementary Figure 6. The similarity matrix for the seizure shown in Supplementary Figure 2. The color code is the same as Supplementary Figure 1

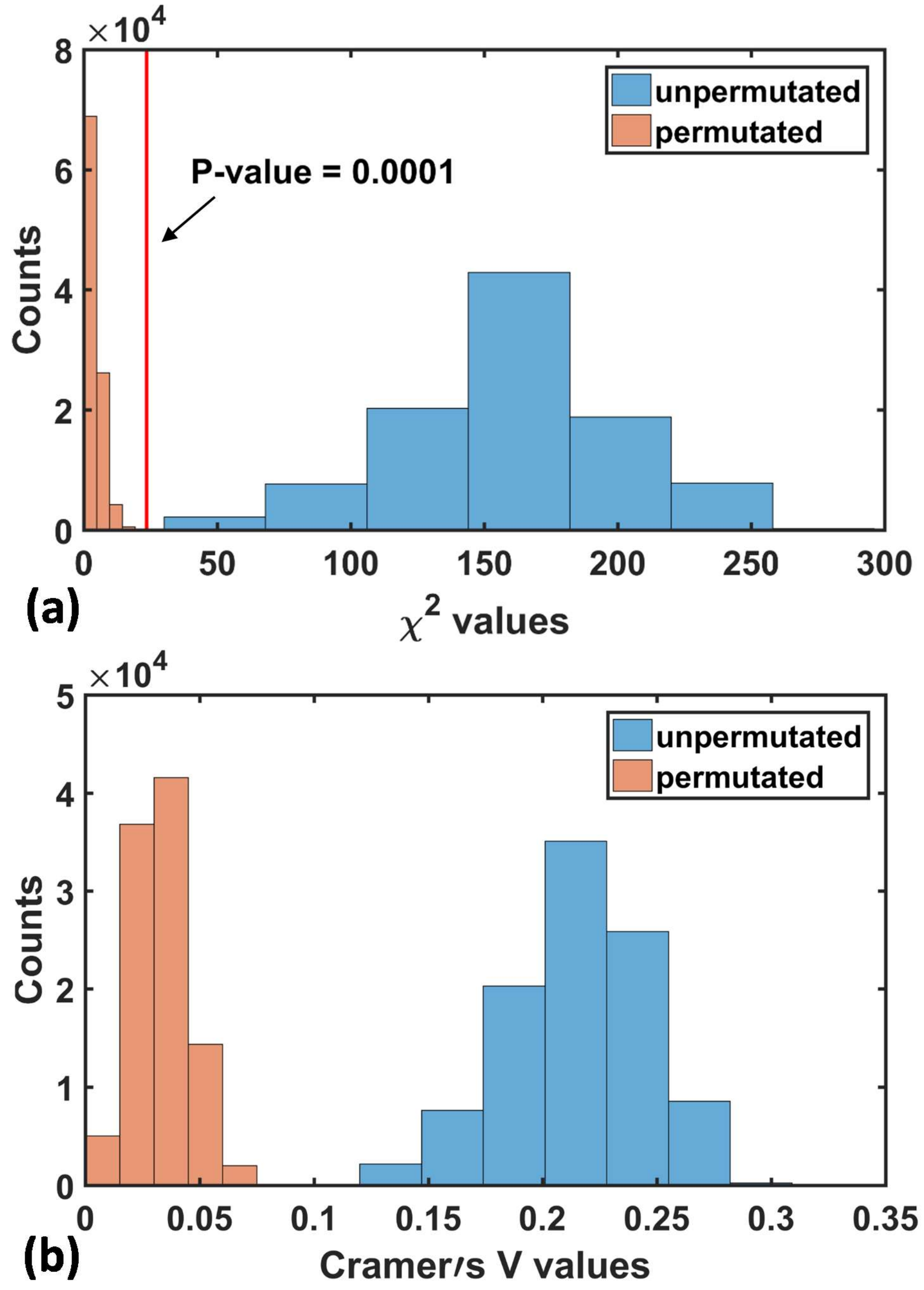

Supplementary Figure 7. (a) The histogram of the obtained χ^2 values after 100,000 runs with different random seeds. The red line indicates the χ^2 value for p-value = 0.0001. On the other hand, permutations on the network states and the propagating types lead to results that fail to reject the null hypothesis. (b) The histogram of the Cramer's V values computed from the above χ^2 values.

Supplementary Movie 1.mp4

Supplementary Movie 1. Representative movie clip showing that the recorded theta oscillations is a globally stationary wave where signals in all the channels rise and fall synchronously with the same phase. The movie is played 10 times slower than reality for better visualization.

Supplementary Movie 2.mp4

Supplementary Movie 2. Representative movie clip showing that during the seizure, different propagating waves emerge, including right-to-left, left-to-right, and spiral waves. Different propagating waves switch between each other during the seizure burst. The movie is played 20 times slower than reality for better visualization.

Supplementary Movie 3.mp4

Supplementary Movie 3. Representative movie clip showing that between the seizures, the majority of the epileptic spikes are right-to-left waves. The movie is played 20 times slower than reality for better visualization.
